# Reduced masticatory stimuli modulate myokine secretion in the masseter muscle in mice

**DOI:** 10.1371/journal.pone.0341417

**Published:** 2026-01-29

**Authors:** Marin Kawasaki, Chiho Kato, Moe Tanigawa, Eri Misawa, Keigo Nakamura, Haruka Inaba, Yasunori Abe, Satoshi Kokai, Takashi Ono

**Affiliations:** Department of Orthodontic Science, Graduate School of Medical and Dental Sciences, Institute of Science Tokyo, Tokyo, Japan; Qassim University, SAUDI ARABIA

## Abstract

Mastication is essential for oral function and systemic metabolic regulation. The impact of soft diets, which reduce masticatory load, on myokine signaling remains unclear. Accordingly, we examined whether reduced mastication alters myokine secretion from the masseter muscle and affects muscle development and systemic metabolic regulation. Male C57BL/6J mice were fed either hard or soft diets for short-term (1 week) or long-term (7 weeks). Body weight, masseter muscle weight, epididymal fat weight, and fiber cross-sectional area were assessed. The expression of key myokines (IL-6, IL-10, TNF-α, Nfkb1, and myostatin [Mstn]) was measured using qPCR (quantitative polymerase chain reaction), and myostatin protein levels were evaluated using immunohistochemical assays. Short-term soft diet feeding did not produce any major morphological changes. However, long-term feeding significantly reduced masseter weight and fiber size, while increasing the amount of epididymal fat, despite an unchanged total body weight. At the molecular level, IL-6 expression was consistently lower in soft diet-fed mice, and IL-10 levels declined further with long-term feeding. In contrast, TNF-α, Nfkb1, and Mstn levels were elevated at both ages. The immunohistochemical assays confirmed increased myostatin protein levels in the masseter under soft-diet conditions. These results suggest that reduced masticatory stimulation remodels the biochemical environment of the masseter muscle, suppressing anabolic and anti-inflammatory signals while enhancing catabolic pathways. These alterations impair muscle growth and promote fat accumulation indicating that masticatory load regulates craniofacial muscle development and systemic metabolism through myokine-mediated mechanisms. Therefore, maintaining adequate mastication during growth may be critical for oral health, body composition, and long-term metabolic homeostasis.

## Introduction

Recent epidemiological studies have suggested that mastication is closely associated with oral and systemic health and the development of systemic diseases. Modern dietary habits have shifted toward the daily consumption of soft, processed, and homogeneous foods, leading to a decrease in masticatory load and meal duration [1,2]. Reduced masticatory load has been associated with an increasing prevalence of malocclusion and craniofacial morphological abnormalities, which have significant consequences for jaw growth in children [3,4]. Mastication is not limited to the initial stages of digestion and it modulates mental and physical health. Masticatory activity has been linked to the regulation of satiety [5] and glucose metabolism [4,6]; conversely, a soft diet has been associated with the development of an obese phenotype through multiple physiological mechanisms. Moreover, reduced mastication during growth has been reported to affect the trigeminal ganglia and impair cognitive function [7]. However, the impact of decreased masticatory load (due to softening of the modern diet) on muscle development and metabolism during growth remains unclear.

Myokines, which are proteins secreted by skeletal muscle fibers, serve autocrine and paracrine functions within the skeletal muscle and mediate systemic crosstalk with organs such as the liver and bone [8]. Myokines have been shown to promote bone formation [9], enhance endothelial function [10], facilitate the development of brown adipocyte-like cells [11], suppress cancer cell proliferation [12], and reduce the risk of type 2 diabetes mellitus [13]. Given their involvement in various physiological processes, alterations in myokine secretion from the masseter muscle resulting from reduced masticatory stimulation may have widespread effects on the systemic physiology. Among these myokines, interleukin-6 (IL-6), a well-known pro-inflammatory cytokine present in both blood and skeletal muscle, is typically secreted by immune cells [14]. However, recent studies have demonstrated that IL-6 released from the skeletal muscle during exercise exhibits anti-inflammatory properties, promoting IL-10 production while inhibiting tumor necrosis factor-α (TNF-α) synthesis [15]. IL-10 triggers changes in the macrophage phenotype that promote muscle growth and regeneration [16]. TNF-α activates the transcription factor NF-κB via oxidative stress, thereby enhancing the degradation of muscle proteins [17]. Under physiological conditions, IL-6 plays a protective role in maintaining muscle mass [18]. Another key regulator of muscle homeostasis is myostatin, a member of the transforming growth factor-β (TGF-β) superfamily, which is predominantly produced in skeletal muscle and acts to suppress muscle growth and promote protein degradation [13]. Myostatin signaling is tightly controlled at multiple levels—including transcriptional and epigenetic regulation and sequestration by extracellular binding proteins [19,20]. Increased myostatin expression has been linked to obesity [13,21]. However, although systemic skeletal muscle responses have been extensively studied, the mechanisms by which these myokines are secreted from the masseter in response to altered masticatory stimulation and their potential systemic effects remain unclear. We hypothesized that, similar to other skeletal muscles, the masseter muscle secretes myokines and influences both local and systemic physiology. This objective of this study was to evaluate how reduced masticatory stimulation alters myokine secretion in the masseter muscle of mice, to clarify its role in muscle morphology, and to explore its potential systemic physiological effects.

## Materials and methods

The study protocol was approved by the Institutional Animal Care and Use Committee of the Institute of Science, Tokyo (A2024-107A). All animal experiments adhered to institutional regulations for animal care and were conducted in line with ARRIVE recommendations.

### Experimental animals

**Fig 1:**
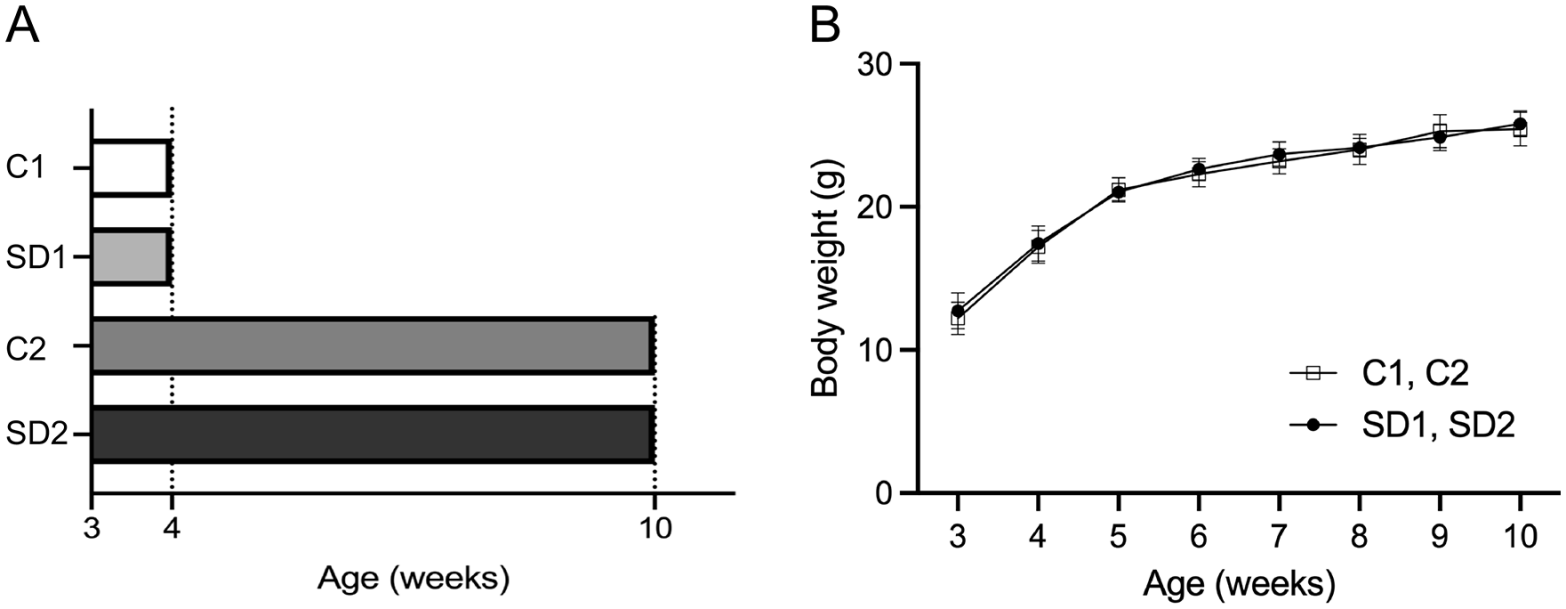
Experimental design and changes in body weight. **(A)** Experimental design showing the feeding protocols for each group: control groups (C1 and C2) were fed a hard diet, whereas soft diet groups (SD1 and SD2) were fed a powdered diet. **(B)** Changes in body weight from weaning age to sacrifice in all groups. No significant differences were observed among the four groups (n = 8 per group). Data are reported as mean ± standard abbreviation (SD). Error bars represent the SD. Abbreviations: C1, control group 1; SD1, Soft Diet 1 group; C2, control group 2; SD2, Soft Diet 2 group.

Three-week-old male C57BL/6J mice were purchased from the Sankyo Labo Service Corporation (Tokyo, Japan). Using simple randomization, the mice were divided into four groups (n = 8 per group; total n = 32): in the short-term treatments, the Control 1 group (C1) was fed a hard diet consisting of chow pellets (CLEA Japan, Tokyo, Japan) from 3 to 4 weeks of age (short-term treatment); the Soft Diet 1 group (SD1) was fed a powdered (soft) diet (CLEA Japan) from 3 to 4 weeks of age. In the long-term treatments, the Control 2 group (C2) was fed chow pellets from 3 to 10 weeks of age, and the Soft Diet group (SD2) was fed only powder from 3 to 10 weeks of age (Fig 1A). The nutritional compositions of the hard and soft diets were identical. Animals were housed in a climate-controlled room with a 12-hour light/dark cycle and had ad libitum access to food and water. The body weights of the mice were measured once a week (Fig 1B). Treatments were initiated simultaneously at 3 weeks of age and all mice were maintained under identical housing conditions. The differences between the short-term (C1 and SD1) and long-term (C2 and SD2) groups, therefore, reflect developmental progression from 4 to 10 weeks of age.

### Sample collection

**Fig 2:**
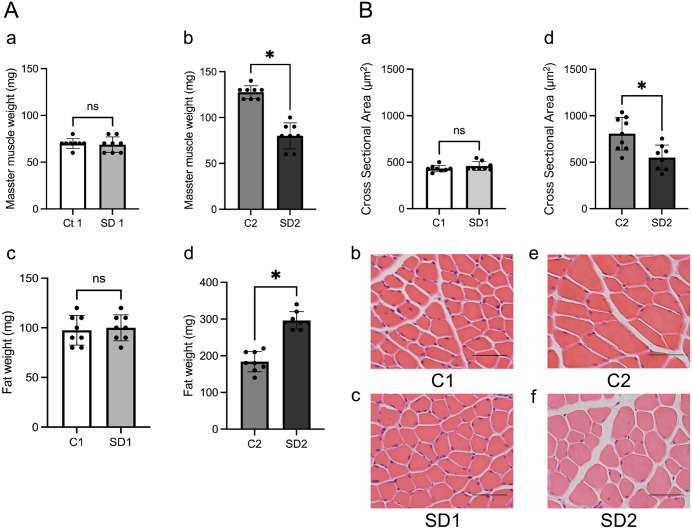
Assessment of the masseter muscle. **(A)** Masseter muscle weight and epididymal fat pad weight in the C1, SD1, C2, and SD2 groups at 4 weeks (a, c) and 10 weeks (b, d) of age (n = 8 per group). **(B)** Quantitative analysis of the masseter muscle fiber cross-sectional area in the C1, SD1, C2, and SD2 groups at 4 weeks (a) and 10 weeks (d) of age (n = 8 per group). Representative hematoxylin and eosin **(H&E)**-stained sections are shown for C1 **(b)**, SD1 **(c)**, C2 **(e)**, and SD2 (f) groups. Data are reported as mean ± SD. *: p < 0.05. Scale bar = 50 µm.

Following euthanasia by isoflurane inhalation, the masseter muscle and epididymal white adipose tissue were excised in accordance with the guidelines of the Institutional Animal Care and Use Committee of the Institute of Science Tokyo. The tissues were washed with physiological saline, blotted dry, and weighed using an electronic balance. Data are reported as mean ± standard deviation (SD) (Fig 2A). Statistical analyses were performed using Student’s t-test, with p < 0.05 considered statistically significant.

### Hematoxylin and eosin (HE) staining

The masseter muscles (superficial and part of the deep masseter muscle) that were fixed in 4% formalin solution for 2 days were cut in cross-section and prepared for examination. Vertical section embedding was performed using paraffin to produce paraffin blocks. This block was cut into 4 μm thick slices, which were then attached to glass slides. After deparaffinization and hydration with xylene and alcohol, the samples were stained with hematoxylin and eosin (H&E). The number of muscle fibers was calculated at 400 × magnification using a light microscope (Nikon ECLIPSE80i, Nikon, Tokyo, Japan), and the average value of the cross-sectional area of the muscle fiber was analyzed using a digital image analyzer (Image J 1.53f51, NIH, Maryland, USA).

### Quantitative reverse transcription polymerase chain reaction

Total RNA was extracted from masseter muscle samples with the RNeasy Plus Universal Mini Kit (QIAGEN, Hilden, Germany) following the manufacturer’s protocol. RNA concentration was quantified with a NanoDrop One spectrophotometer (Thermo Fisher Scientific, Waltham, MA, USA) and normalized to 50 ng/µL. cDNA was generated from total RNA by reverse transcription with the PrimeScript RT Master Mix (Perfect Real Time, #RR036A, Takara, Kusatsu, Japan) in accordance with the manufacturer’s protocol. Quantitative RT polymerase chain reaction (qPCR) was conducted using an Applied Biosystems 7500 Real-Time PCR System (Thermo Fisher Scientific). PCR amplification consisted of 60 cycles of denaturation at 95°C for 3 s and annealing/extension at 60°C for 30 s using IL-6 primers (Mm00446190_m1; Thermo Fisher Scientific), IL-10 primers (Mm01288386_m1; Thermo Fisher Scientific), Tnf-α primers (Mm00443258_m1; Thermo Fisher Scientific), Nfkb1 primers (Mm00476361_m1; Thermo Fisher Scientific), myostatin primers (Mm01254559_m1; Thermo Fisher Scientific), and glyceraldehyde 3-phosphate dehydrogenase (Gapdh) primers (Mm99999915_g1; Thermo Fisher Scientific). The results were analyzed using the comparative Ct method and normalized to the expression of Gapdh. Statistical analyses for qPCR data were performed on ΔCt values, whereas 2^ − ΔΔCt values were used only for graphical representation. All qPCR measurements were conducted in technical triplicates to ensure reproducibility and minimize pipetting variability and the mean Ct value was used for analysis. There were eight biological replicates per group.

### Immunohistochemical staining

The masseter muscle was fixed in 4% formalin for 2 days, embedded in paraffin, sectioned to a thickness of 4 μm, and mounted on a glass slide. After deparaffinization with xylene and rehydration with ethanol, endogenous peroxidase activity was blocked by immersion in 3% hydrogen peroxide for 15 min. Following washing with PBS (Phosphate-buffered saline), the sections were incubated overnight at 4°C with rabbit polyclonal anti-myostatin antibody (ab203076, Abcam, Cambridge, UK) diluted in PBS containing 0.1% BSA (Bovine Serum Albumin). Subsequently, secondary antibody incubation and ABC reagent processing were performed using the VECTASTAIN Elite ABC-HRP Kit (Vector Laboratories, USA), and color development was performed using DAB (diaminobenzidine) substrate for 10 min. Counterstaining was performed using Mayer’s hematoxylin, followed by dehydration and mounting. To evaluate myostatin protein expression in each masseter muscle, myostatin-immunopositive areas were observed under a light microscope (Nikon ECLIPSE 80i; Nikon, Tokyo, Japan). For each sample, one section was analyzed at 400 × magnification in six fields of view, each measuring 300 × 300 pixels (0.05 × 0.05 mm). All procedures for image acquisition, identification, and processing were standardized before image capture. Six fields per section were randomly selected. Immunohistochemically positive regions were extracted using a digital image analyzer (Image J 1.53f51, NIH, Maryland, USA), and the proportion of anti-myostatin antibody-positive area was calculated by dividing the positive area by the total masseter muscle area in each stained image [22]. The proportion of myostatin-immunopositive area (“myostatin area fraction”) was calculated as the ratio of the positively stained area to the total muscle area in each image, using threshold-based pixel segmentation in ImageJ. This value represents the fraction of area that was positive for myostatin staining rather than the staining intensity. Each biological replicate corresponded to one animal (n = 8 per group). Immunohistochemical analysis was performed on one section per animal and six randomly selected fields (300 × 300 μm) were quantified using ImageJ. The average value from the six fields was treated as one technical replicate for each animal.

### Statistical analyses

All data are reported as mean ± SD. Comparisons of masseter muscle weight, muscle fiber cross-sectional area, epididymal fat weight, and immunohistochemical expression of myostatin between the two groups were performed using Student’s t-test. For comparisons across groups and time points (4 and 10 weeks), a two-way analysis of variance was conducted, followed by Sidak’s multiple comparisons test when appropriate. When the assumption of normality was not satisfied, the Mann–Whitney U test was used. Statistical analyses were performed using GraphPad Prism (version 9.3.0; GraphPad Software, USA), and a p-value <0.05 was considered statistically significant.

## Results

### Evaluation of masseter muscle mass, muscle fibers, and epididymal fat

To investigate the time-dependent effects of a soft diet on the masseter muscle and epididymal fat pad, we measured the weights of these tissues from mice fed either a hard or soft diet and euthanized at either 4 weeks of age (short-term treatment) or 10 weeks (long-term treatment) of age. No significant differences in body weight were observed among the four groups throughout the experimental period (Fig 1B). At 4 weeks of age, there was no statistically significant difference in the masseter muscle and epididymal fat weights between the C1 and SD1 groups. In contrast, at 10 weeks of age, the SD2 group showed significantly (p-value less than 0.05) lower masseter muscle weight and higher epididymal fat than the corresponding results in the C2 group (Fig 2A). H&E staining of masseter muscle sections was performed to assess the average muscle fiber cross-sectional area. Consistent with the muscle weight results, no significant difference was observed in the fiber area between the C1 and SD1 groups at 4 weeks of age. However, the SD2 group (10 weeks of age) showed a significantly (p < 0.05) smaller average muscle fiber area than the corresponding control group (C2) (Fig 2B). These findings indicate that short-term soft diet feeding does not alter muscle morphology, whereas long-term soft diet consumption leads to atrophy of the masseter muscle and increased fat accumulation.

### Hypertrophy-related myokines

**Fig 3:**
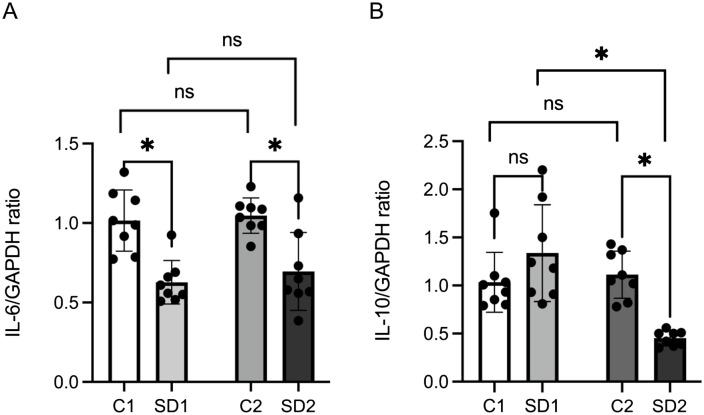
mRNA expression levels of IL-6 and IL-10. mRNA expression levels of (a) IL-6 and (b) IL-10 in tissues at 4 and 10 weeks, measured using quantitative real-time PCR (qPCR). The expression levels were normalized to GAPDH and calculated using the ΔΔCt method. Data are reported as mean ± SD (n = 8 per group). *: p < 0.05. GAPDH, glyceraldehyde 3-phosphate dehydrogenase; IL-6, interleukin-6.

To clarify the molecular mechanisms underlying these observations, we analyzed the expression of hypertrophy-associated myokines IL-6 and IL-10 in the masseter muscle at 4 and 10 weeks of age. IL-6 expression was lower in SD1 than in C1 and in SD2 than in C2; however, no significant differences were detected between C1 and C2 or between SD1 and SD2 (Fig 3a). Regarding IL-10 levels, no significant difference was observed between the C1 and SD1 groups at 4 weeks. However, at 10 weeks, the SD2 group showed significantly lower IL-10 expression than the C2 group. Although no significant difference was observed between C1 and C2, SD2 group showed a significantly (p < 0.05) lower value than the SD1 group (Fig 3b). These results suggest that long-term soft diet consumption leads to a decrease in the expression of hypertrophy-related myokines in the masseter muscle, potentially impairing muscle growth and homeostasis.

### Atrophy-related myokines

**Fig 4:**
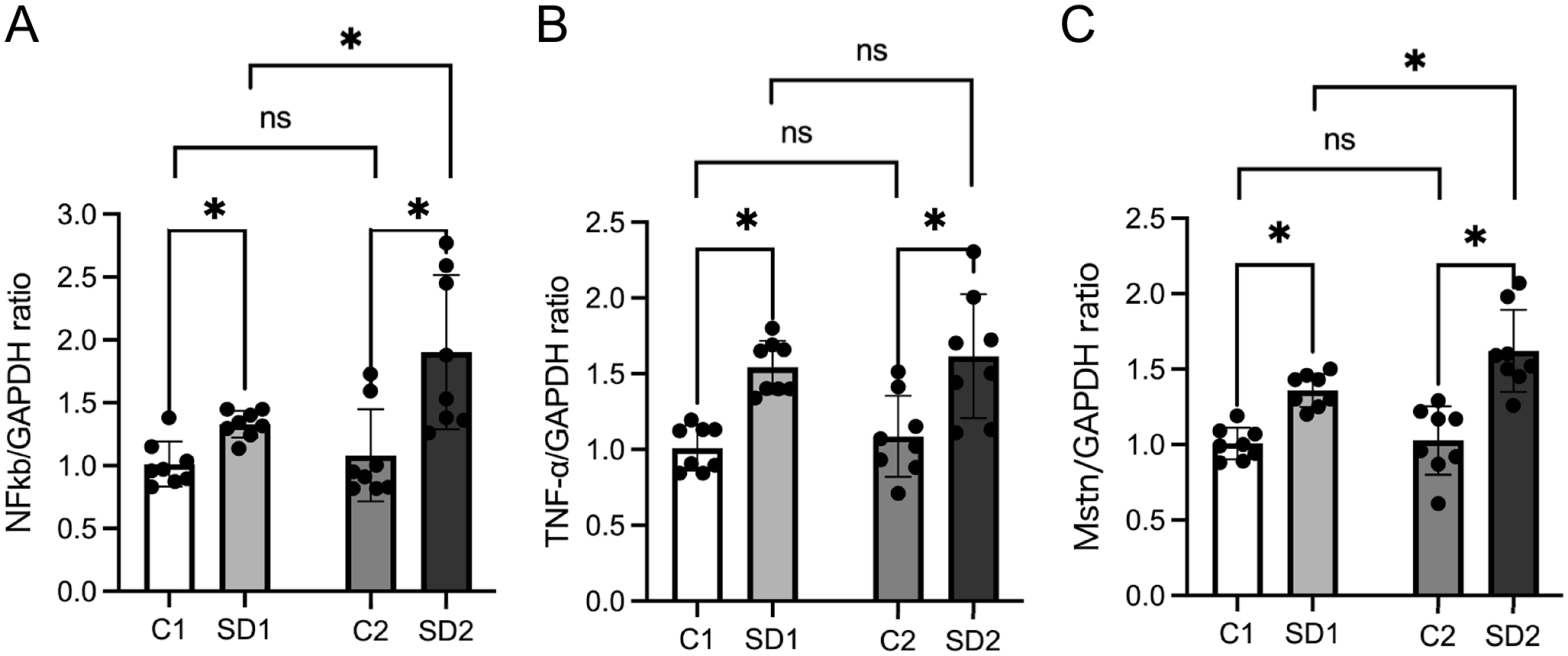
mRNA expression levels of TNF-α, NF-κB, and myostatin. mRNA expression levels of (a) TNF-α, (b) NF-κB, and (c) myostatin in tissues at 4 and 10 weeks, measured using quantitative real-time PCR (qPCR). The expression levels were normalized to GAPDH and calculated using the ΔΔCt method. Data are reported as mean ± SD (n = 8 per group). *: p < 0.05. TNF-α: tumor necrosis factor-α, NF-κB: nuclear factor kappa-light-chain-enhancer of activated B cells.

To investigate the expression of myokines associated with muscle atrophy, we analyzed the expression of TNF-α, NF-κB, and Myostatin in the masseter muscle at 4 and 10 weeks of age. All three myokines showed higher expression in SD1 and SD2 compared with the corresponding levels in C1 and C2. Although no significant differences were observed between C1 and C2, SD2 exhibited a significantly (p < 0.05) lower expression than SD1 (Fig 4a–c). In the case of TNF-α and NF-κB, no significant differences were observed between C1 and C2 or between SD1 and SD2. In contrast, myostatin expression did not differ between C1 and C2, but was significantly (p < 0.05) higher in SD2 than in SD1 (Fig 4c). These findings indicate that reduced masticatory stimulation promotes the expression of atrophy-related myokines, which may contribute to the suppression of masseter muscle development.

### Quantitative immunohistochemical analysis of myostatin expression in masseter muscle

**Fig 5:**
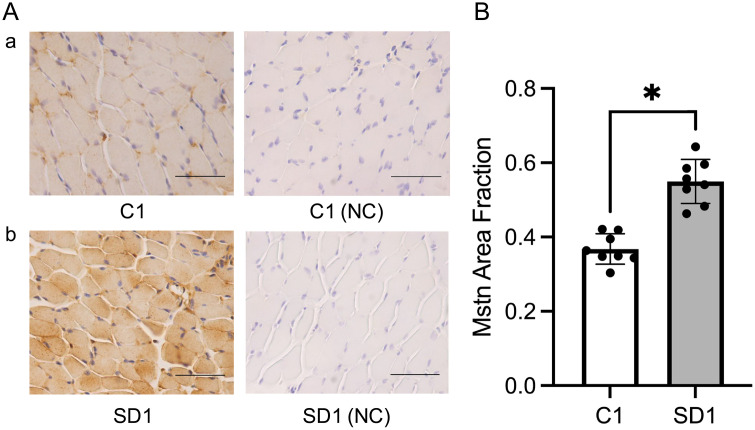
Immunohistochemical analysis of myostatin expression in the C1 and SD1 groups. **(A)** Representative immunohistochemical staining of myostatin in masseter muscle sections from C1 **(a)** and SD1 **(b)**. Negative controls (NC; omission of primary antibody) are shown. Positive immunoreactivity is indicated using brown cytoplasmic staining. The sections were counterstained with hematoxylin. Images were captured at 400 × magnification. Scale bar = 50 μm. **(B)** Quantitative analysis of the myostatin-positive area using ImageJ. Data are reported as mean ± SD (n = 8 per group). *: p < 0.05.

**Fig 6:**
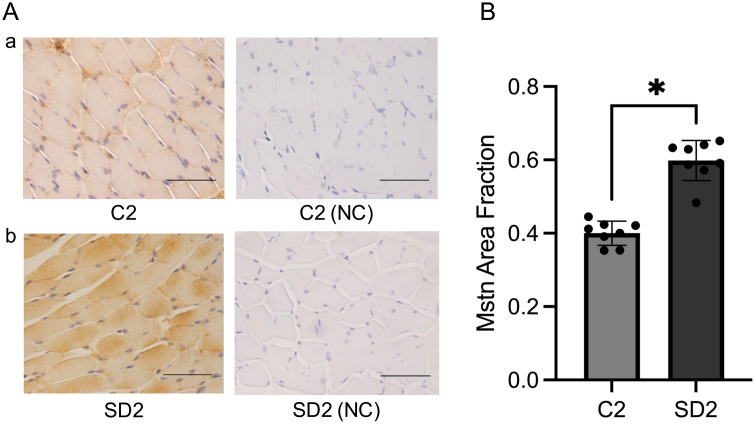
Immunohistochemical analysis of myostatin expression in the C2 and SD2 groups. **(A)** Representative immunohistochemical staining of myostatin in masseter muscle sections from C2 **(a)** and SD2 **(b)**. Negative controls (NC; omission of primary antibody) are shown. Positive immunoreactivity is indicated using brown cytoplasmic staining. Sections were counterstained with hematoxylin. Images were captured at 400 × magnification. Scale bar = 50 μm. **(B)** Quantitative analysis of the myostatin-positive area using ImageJ. Data are reported as mean ± SD (n = 8 per group). *: p < 0.05.

To evaluate the protein expression levels of myostatin, we performed immunohistochemical staining of the masseter muscle of mice at 4 and 10 weeks of age. Myostatin expression was significantly (p < 0.05) higher in SD1 than in C1 and in SD2 than in C2 at both 4 and 10 weeks of age (Figs 5, 6). These results confirm that myostatin expression is elevated in the masseter muscle under soft-diet conditions at the protein level, consistent with the gene expression data, thereby supporting the increased expression of myostatin.

## Discussion

In this study, we investigated the effects of reduced masticatory stimulation during the growth period in mice on the myokine secretion from the masseter muscle and the potential local and systemic consequences of such alterations. The results demonstrated that reduced masticatory stimulation during growth induced dysregulation of myokine secretion in the masseter muscle, leading to impaired muscle development. Furthermore, changes in myostatin expression and epididymal fat mass suggest a relationship between reduced masticatory stimulation and increased fat accumulation. Although food intake was not quantitatively measured in this study, both diets were nutritionally identical and all animals were maintained under ad libitum feeding conditions. Moreover, there were no significant differences in overall body weight among the groups (Fig 1B), indicating that the effects of the soft diet were not related to general body growth or caloric intake but were specific to the masseter muscle weight and epididymal fat weight. These findings provide insights into the interactions between masticatory function, muscle-derived signaling molecules, and systemic metabolic regulation.

To examine the effects of different durations of soft diet intake on masseter muscle development, we evaluated the muscle fiber cross-sectional area and masseter muscle weight. One week of soft diet feeding did not produce significant changes in either parameter. In contrast, long-term feeding for 7 weeks significantly suppressed the muscle development. An increase in skeletal muscle mass occurs when the rate of muscle protein synthesis exceeds that of protein degradation [23,24]. This balance promotes protein accumulation, leading to muscle fiber hypertrophy and increased muscle mass during growth. Although high-resistance loading contributes to muscle hypertrophy, the physiological mechanisms—particularly how mechanical stimuli influence protein synthesis signaling—remain incompletely understood [25]. The results of the current study suggest that short-term (1 week) soft diet intake does not significantly affect protein metabolism via reduced mechanical loading, whereas sustained load reduction over 7 weeks may impair muscle growth through decreased protein synthesis activity and/or enhanced degradation.

IL-6, present in both blood and skeletal muscle, is considered a pro-inflammatory cytokine secreted at high levels by immune cells [14]. However, recent studies have shown that exercise-induced IL-6 secreted by muscle cells exerts anti-inflammatory effects similar to those of growth factors [15]. IL-6 promotes the production of IL-1 receptor antagonist and IL-10 while suppressing the synthesis of the pro-inflammatory cytokine TNF-α [15,26]. TNF-α has been reported to enhance signaling, including that of myostatin, via the activation of the redox-sensitive transcription factor NF-κB, thereby promoting muscle protein degradation and reducing muscle function [17,27]. In the current study, although no significant differences in masseter muscle weight or fiber cross-sectional area were observed between the control and soft diet groups at 4 weeks of age, the SD group showed reduced IL-6 expression and increased TNF-α, NF-κB, and Myostatin expression. This suggests that even before morphological changes become apparent, reduced masticatory stimulation during growth may shift the molecular environment toward a catabolic state, making the muscles more susceptible to subsequent atrophy. At 10 weeks of age, IL-6 expression remained reduced in the SD group, whereas IL-10 expression decreased. IL-6 and IL-10 play important roles in muscle regeneration and homeostasis, and their sustained reduction may impair muscle maintenance and adaptive growth [15]. These changes suggest that the long-term absence of masticatory stimulation may lead to sustained suppression of anti-inflammatory and growth-promoting signals. The current findings suggest that masticatory stimulation is essential as a source of mechanical load and for maintaining an environment in developing muscles characterized by sufficient IL-6 and IL-10 expression and suppression of TNF-α, NF-κB, and Myostatin.

Myostatin, a secreted signaling molecule belonging to the activin/TGF-β/bone morphogenetic protein family, is a negative regulator of skeletal muscle growth [28]. By binding to activin receptor type II family members, particularly activin receptor type IIB, myostatin inhibits cell proliferation, differentiation, and protein synthesis in skeletal muscles, thereby regulating muscle growth [29,30]. Overexpression of myostatin has been reported to be induced by the pro-inflammatory cytokine TNF-α via the activation of NF-κB [27]. Moreover, myostatin is a regulator of muscle size and systemic metabolic regulator, as it is involved in lipid metabolism [31,32], glucose metabolism [33], and even the promotion of atherosclerosis [34]. In the current study, the soft diet group showed increased TNF-α, NF-κB, and Myostatin expression in the masseter muscle, consistent with the activation of catabolic signaling due to reduced mechanical load. These molecular changes were observed in conjunction with an increased epididymal fat mass, despite no differences in the total body weight. In this study, regardless of the feeding duration, the expression of TNF-α, NF-κB, and Myostatin in the masseter muscle was higher in the soft diet groups than in the control groups at both 4 and 10 weeks of age. Furthermore, immunohistochemical analysis revealed that myostatin protein expression in the masseter muscle was elevated in the soft diet groups under both short- and long-term feeding conditions. These findings suggest that the upregulation of myostatin expression in the masseter muscle, concomitant with the activation of catabolic signaling due to reduced mechanical load, may contribute to the local inhibition of muscle development and systemic fat accumulation through endocrine pathways. Moreover, given that myostatin is associated with metabolic diseases, such as diabetes and cardiovascular disorders, changes in its expression resulting from reduced masticatory function could have widespread long-term health implications.

In the current study, we observed that feeding mice a soft diet led to dysregulation of myokine secretion. One week of soft diet feeding did not affect the masseter muscle or epididymal fat; however, 7 weeks of a soft diet resulted in suppressed development of the masseter muscle and increased epididymal fat in the soft-diet group. Myokines exert endocrine effects on distant organs. These results provide new insights into the impact of the recent trend toward soft diets on masseter muscle development and systemic physiology from the perspective of myokine regulation. Diets that ensure adequate masticatory stimulation are considered essential for maintaining the masseter muscle at an optimal level. Furthermore, this study demonstrated that masticatory stimulation contributes to maintaining an appropriate balance of myokines, thereby promoting proper development of the masseter muscle. Masseter muscle thickness is greatest in Class I normal occlusion, decreases progressively in Class I, Class II division I, and Class II division II malocclusions, and is lowest in Class III malocclusion [35]. Therefore, insufficient development of the masseter muscle due to inadequate masticatory stimulation during growth may compromise occlusal stability and affect the facial morphology. Based on these findings, oral management from early childhood and orthodontic treatment to improve masticatory function in children are crucial for masseter muscle development and the acquisition and maintenance of normal occlusion.

This study has some limitations. First, although the use of a mouse model enabled the strict control of experimental conditions, species differences in craniofacial and muscle physiology limit direct extrapolation of the findings to humans. Second, analyses were limited to two time points—4 and 10 weeks of age—which may not fully reflect the temporal progression of molecular and morphological changes. Third, although reduced masticatory stimulation was associated with altered myokine secretion, direct mechanistic evidence linking these changes to reduced masseter development, increased fat accumulation, or systemic effects was not obtained. Further studies are needed to examine intracellular signaling pathways such as mTOR [36] and proteolytic systems including Atrogin-1 and MuRF1 [37] and investigate molecular responses in other skeletal muscles and circulating myokines. Additionally, future studies using immune-cell markers such as F4/80 or CD68 will be required to determine whether the altered myokine expression originates from muscle fibers or infiltrating immune cells.

## Conclusions

This animal study demonstrated that soft diet feeding, which reduces masticatory stimulation, was associated with alterations in myokine secretion from the masseter muscle. These changes in myokine expression may affect local muscle tissue and systemic physiological functions, such as body-fat accumulation. However, as these findings are based on an animal model and correlative analyses, further mechanistic studies are required to determine the causal pathways underlying these associations. These observations highlight the potential importance of maintaining proper oral function and suggest the role of early orthodontic intervention during childhood in promoting the healthy development of facial muscles and normal systemic growth and metabolic regulation.

## Supporting information

S1 File Data. (XLSX)
